# Crystal structure of [*t*BuMgCl]_2_[MgCl_2_(Et_2_O)_2_]_2_

**DOI:** 10.1107/S2056989023002190

**Published:** 2023-03-15

**Authors:** Maurice Metzler, Michael Bolte, Matthias Wagner, Hans-Wolfram Lerner

**Affiliations:** a Institut für Anorganische und Analytische Chemie, Goethe-Universität Frankfurt, Max-von-Laue-Str. 7, 60438 Frankfurt/Main, Germany; Vienna University of Technology, Austria

**Keywords:** crystal structure, Grignard reagent, open-cube cluster

## Abstract

The title compound, di-μ_3_-chlorido-tetra-μ_2_-chlorido-tetra­kis­(diethyl ether-κ*O*)bis­(1,1-di­methyl­eth­yl)tetra­magnesium, [Mg_4_(C_4_H_9_)_2_Cl_6_(C_4_H_10_O)_4_], features an Mg_4_Cl_6_ open-cube cluster. The two four-coordinate Mg^2+^ ions show an almost tetra­hedral coordination, whereas the two six-coordinate Mg^2+^ ions have their ligands in an octa­hedral environment. The Mg—Cl bond lengths differ depending on the coordination number (2 or 3) of the bridging μ-Cl^−^ ligands. There are few comparable structures deposited in the Cambridge Structural Database.

## Abstract 2

The crystal structure of [*t*BuMgCl]_2_[MgCl_2_(Et_2_O)_2_]_2_ features an Mg_4_Cl_6_ open-cube cluster with both four- and six-coordinate Mg^2+^ ions. The Cl^−^ ions adopt bridging positions. Inter­molecular C—H⋯Cl hydrogen bonds link adjacent units of the title compound into chains extending parallel to [010].

## 1. Chemical context

**Figure 1:**
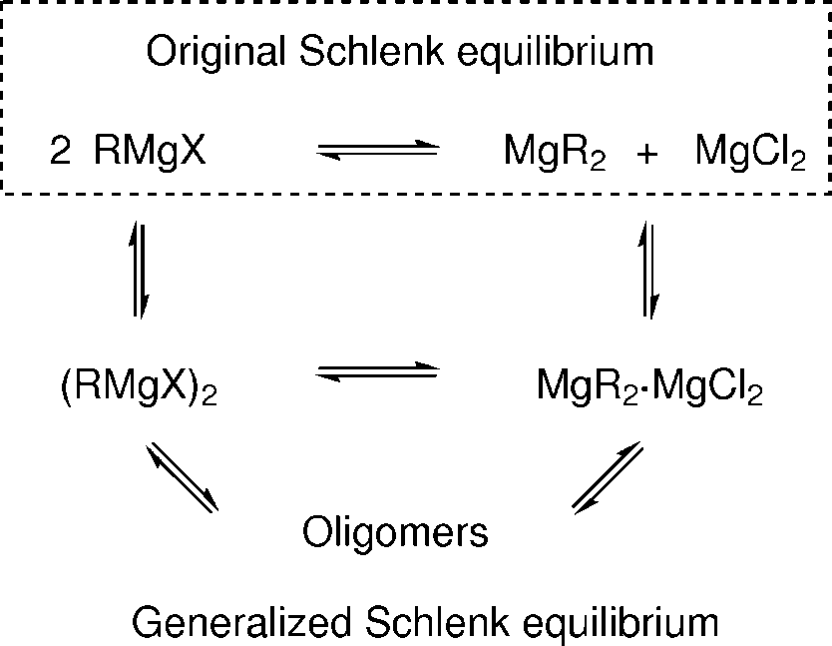
Original and generalized Schlenk equilibrium (solvent mol­ecules neglected; Peltzer *et al.*, 2017 ▸).

Grignard reagents *(R*Mg*X*) are among the most commonly used organometallic reagents in synthesis. However, their mol­ecular structures are highly diverse and therefore subject to ongoing research (Elschenbroich, 2008 ▸; Peltzer *et al.*, 2020 ▸; Curtis *et al.*, 2020 ▸). The structures of *R*Mg*X* in solution depend on the nature of the solvent, the bulkiness of the organic moiety *R*, and the choice of the halide *X* (Peltzer *et al.*, 2017 ▸). Moreover, the Schlenk equilibrium can convert *R*Mg*X* into Mg*R*_2_ and Mg*X*_2_ (Schlenk & Schlenk jun., 1929 ▸). The formation of halide bridges between the Lewis-acidic Mg^2+^ ions (Mg—*X*—Mg) allows for dimeric and oligomeric structures that are in equilibrium with their monomeric units (Fig. 1 ▸). Further coordination sites at Mg^2+^ are often saturated by donor-solvent mol­ecules (Seyferth, 2009 ▸).

**Figure 2:**
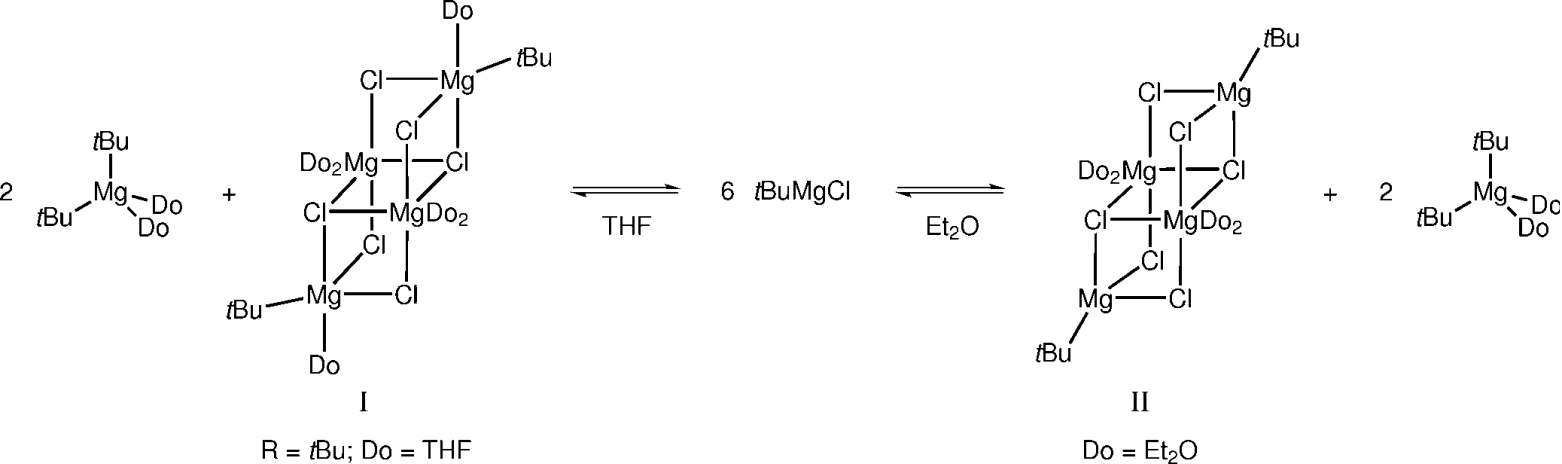
Open-cube structures like **I** (*R* = ^*t*^Bu) are obtained by crystallization of ^*t*^BuMgCl from THF solutions, whereas the less-solvated title compound **II** crystallizes from Et_2_O solutions of ^*t*^BuMgCl.

Since the analysis of Grignard reagents in solution is challenging, X-ray crystallography has emerged as an alternative, frequently used method to investigate their mol­ecular compositions. A recurring structural motif in the solid state is the open-cube cluster [*R*MgCl(THF)]_2_[MgCl_2_(THF)_2_]_2_ (**I**; *R* = Me, Et, ^*i*^Pr, ^*n*^Bu, ^*t*^Bu). Toney & Stucky (1971 ▸), Sakamoto *et al.* (2001 ▸), as well as our group (Blasberg *et al.*, 2012 ▸) provided evidence for such structures obtained by crystallization of *R*MgCl from THF. According to the Schlenk equilibrium, the formation of **I** can be rationalized by assuming aggregation of two *R*MgCl·MgCl_2_ entities. The two independent Mg^2+^ ions in **I** exhibit five- and six-coordination, respectively. We now report [^*t*^BuMgCl]_2_[MgCl_2_(Et_2_O)_2_]_2_ (**II**) as the first example of such open-cube clusters obtained from Et_2_O. It is noteworthy that, unlike those in **I**, the reactive Mg^2+^ ions in the title compound **II** are four-coordinate and, surprisingly, no solvent coordinates to these ^*t*^BuMgCl_3_ entities. Subtle changes such as exchanging THF for the weaker donor Et_2_O seem to have a significant effect on the observed structural motifs (Fig. 2 ▸). 
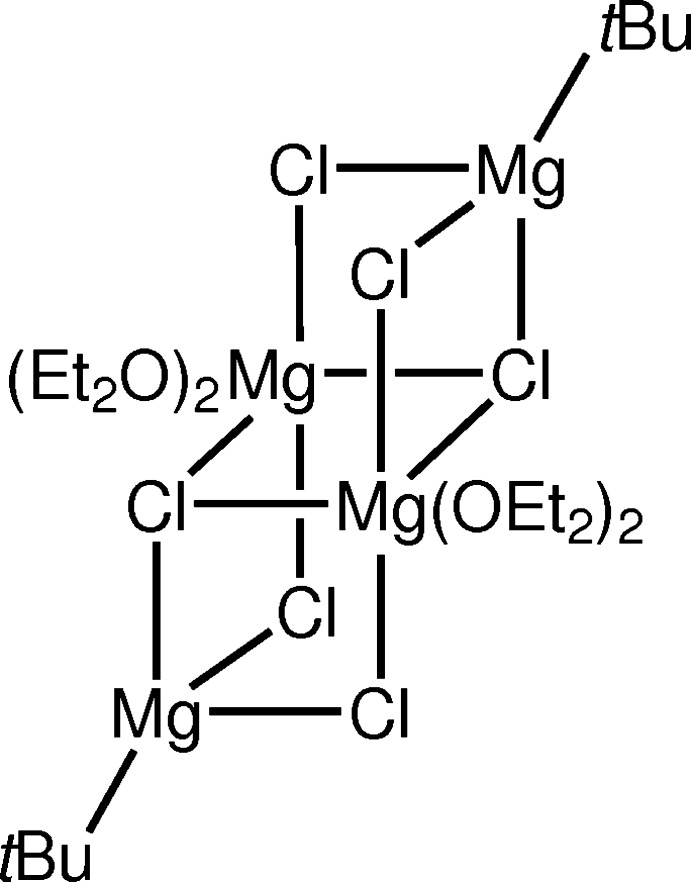


## 2. Structural commentary

**Figure 3:**
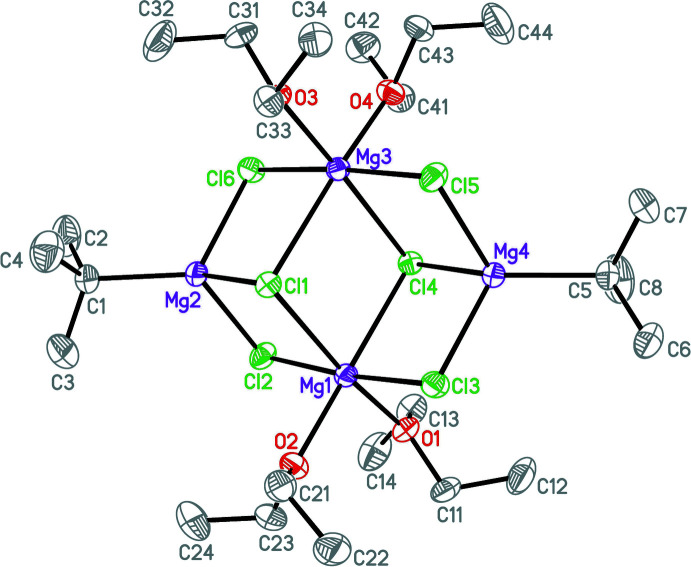
Mol­ecular structure of the title compound. Displacement ellipsoids are drawn at the 50% probability level. Hydrogen atoms are omitted for clarity.

The title compound **II** features an open-cube cluster consisting of Mg^2+^ and Cl^−^ ions (Fig. 3 ▸). The Mg^2+^ ions (Mg1, Mg3) in the Mg_2_Cl_2_ plane are six-coordinate with four Cl^−^ ligands and the O atoms of two Et_2_O mol­ecules in an almost perfect octa­hedral mode. The Mg—Cl distances to the three-coordinate μ_3_-Cl^−^ ligands (Cl1, Cl4) are significantly longer [2.6204 (7)–2.6629 (7) Å] than the Mg—Cl distances to the two-coordinate μ_2_-Cl^−^ ligands (Cl2, Cl3, Cl5, Cl6) [2.4555 (7)–2.4676 (7) Å]. The other two Mg^2+^ ions (Mg2, Mg4) are four-coordinate with three Cl^−^ ligands and one *tert*-butyl group featuring a distorted tetra­hedron. As a result of the geometric strain in these MgCl entities, the Cl—Mg—Cl angles are smaller than the Cl—Mg—C angles. Again, a difference in the Mg—Cl bond lengths can be observed: as expected, the bonds between Mg^2+^ and the μ_3_-Cl^−^ ligands are longer [2.4687 (7) and 2.4689 (7) Å] than the bonds between Mg^2+^ and the μ_2_-Cl^−^ ligands [2.3785 (7)–2.3905 (7) Å].

## 3. Supra­molecular features

**Figure 4:**
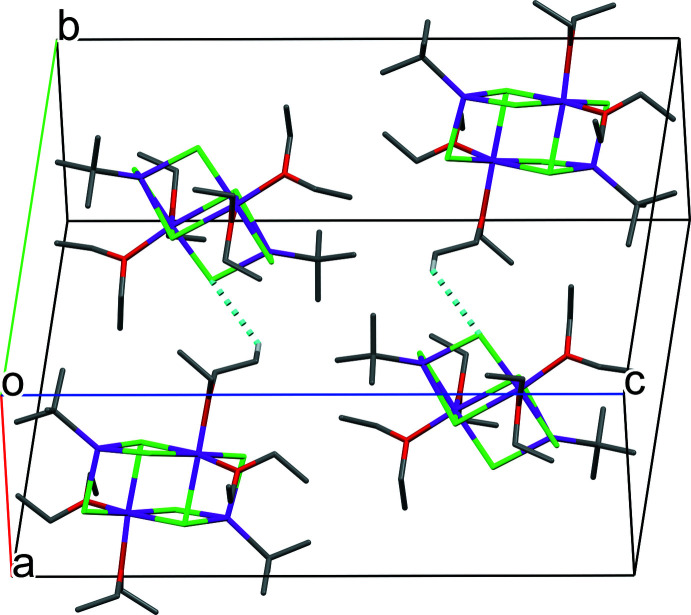
Packing diagram of the title compound **II** showing the C—H⋯Cl hydrogen bonds (cyan) between adjacent mol­ecules of **II**. H atoms not involved in hydrogen bonding are omitted for clarity.

There are two short C—H⋯Cl contacts bridging adjacent mol­ecules of the title compound **II**. These hydrogen bonds (Table 1 ▸) lead to the formation of chains extending parallel to [010]. A packing diagram showing one unit cell is depicted in Fig. 4 ▸. There are no other remarkable inter­molecular inter­actions.

## 4. Database survey

Six comparable structures with a similar Mg_4_Cl_6_ open-cube cluster have been found in the Cambridge Structural Database (version 5.43, update of September 2022; Groom *et al.*, 2016 ▸), *viz.*, [EtMgCl(THF)]_2_[MgCl_2_(THF)_2_]_2_ (MGCLTF; Toney & Stucky, 1971 ▸), [MeMgCl(THF)]_2_[MgCl_2_(THF)_2_]_2_ (QUJSUJ; Sakamoto *et al.*, 2001 ▸), [^*t*^BuMgCl(THF)]_2_[MgCl_2_(THF)_2_]_2_ (QUJTAQ; Sakamoto *et al.*, 2001 ▸), [benzylMgCl(THF)]_2_[MgCl_2_(THF)_2_]_2_ (QUJTEU; Sakamoto *et al.*, 2001 ▸), [^*i*^PrMgCl(THF)]_2_[MgCl_2_(THF)_2_]_2_ (SEJZUE; Blasberg *et al.*, 2012 ▸), and [Me_2_NCH_2_CH_2_CH_2_MgCl]_2_[MgCl_2_(THF)_2_]_2_ (WILMIN; Casellato & Ossola, 1994 ▸). A seventh structure [^*n*^BuMg_3_Cl_5_(THF)_4_]_2_ (ZIHQEO; Pirinen *et al.*, 2013 ▸) also features an open-cube cluster; however, here the reactive Mg^2+^ ions are not part of the cubes. The latter structure is therefore not included in the comparison. Inter­estingly, all the above structures from the database show crystallographic centrosymmetry, with all of them being located at a center of inversion. The title compound, on the other hand, does not show any crystallographic symmetry, although it would be possible for **II** to comply with a crystallographic inversion center. A fundamental difference between the structure of the title compound and the published structures is the coordination sphere of the reactive Mg^2+^ ions. In all structures retrieved from the CSD, these Mg^2+^ ions are five-coordinate and the ligands form a distorted trigonal bipyramid. The calculated geometry indices *τ*_5_ (0.65–0.84) show a varying degree of deviation from the ideal trigonal bipyramidal geometry (τ_5_ = 1; Addison *et al.*, 1984 ▸). The Mg⋯Cl distances to the μ_3_-Cl^−^ ligands in the central Mg_2_Cl_2_ plane (Table 2 ▸) are significantly longer (mean value 2.806 Å) than in **II** (mean value 2.4688 Å), but in between the sum of van der Waals radii (Σr(vdW)[Mg,Cl] = 3.48 Å) and effective ionic radii (Σr(ion)[Mg^2+^,Cl^−^] = 2.47 Å) (Bondi, 1964 ▸; Shannon, 1976 ▸). Nevertheless, the sums of equatorial angles Σθ_eq_ (mean value 358.6°) indicate that the Mg^2+^ ions are five-coordinate in a trigonal bipyramidal mode and that inter­actions with the μ_3_-Cl^−^ ions in the Mg_2_Cl_2_ planes exist. Structures with other halogens than Cl were not found.

## 5. Synthesis and crystallization

Magnesium turnings (9.74 g, 401 mmol, 1.7 eq.) were placed in a Schlenk flask and dried under vacuum by heating. Dry Et_2_O (40 ml) was added to the flask and a solution of ^*t*^BuCl (21.3 g, 230 mmol, 1.0 eq.) in Et_2_O (20 ml) was added dropwise at such a rate as to maintain a gentle reflux (approx. 1 h). After cooling to room temperature, the Grignard solution was separated from residual Mg turnings by cannula transfer into a new Schlenk flask. Upon concentration of the solution at room temperature, colorless crystals of [^*t*^BuMgCl]_2_[MgCl_2_(Et_2_O)_2_]_2_ formed, which were suitable for single-crystal X-ray structure determination.

## 6. Refinement

Crystal data, data collection, and structure refinement details are summarized in Table 3 ▸. H atoms were geometrically positioned and refined using a riding model with C_methyl­ene_—H = 0.99 Å and *U*(H) = 1.2*U*_eq_(C) or with C_meth­yl_—H = 0.98 Å and *U*(H) = 1.5*U*_eq_(C).

## Supplementary Material

Crystal structure: contains datablock(s) I, global. DOI: 10.1107/S2056989023002190/wm5675sup1.cif

Structure factors: contains datablock(s) I. DOI: 10.1107/S2056989023002190/wm5675Isup2.hkl

CCDC reference: 2246966

Additional supporting information:  crystallographic information; 3D view; checkCIF report

## Figures and Tables

**Table 1:** Hydrogen-bond geometry (Å, °)

*D*—H⋯*A*	*D*—H	H⋯*A*	*D*⋯*A*	*D*—H⋯*A*
C32—H32*A*⋯Cl3^i^	0.98	2.96	3.876 (2)	155
C34—H34*A*⋯Cl6^ii^	0.98	2.92	3.602 (2)	127

Symmetry codes: (i) 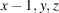 ; (ii) 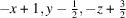 .

**Table 2:** Comparison of Mg⋯Cl distances (Å) and sums of equatorial angles Σθ_eq_ (°) of the five-coordinate Mg^2+^ ions in the literature phases There are two rows for the title compound because it does not show any symmetry, whereas all structures retrieved from the database are located at a center of inversion. Mg⋯μ_2_-Cl: bond lengths are between the five-coordinate Mg^2+^ ions and the μ_2_-Cl^−^ ions. Mg⋯μ_3_-Cl: bond lengths are between the five-coordinate Mg^2+^ ion and the μ_3_-Cl^−^ ion in the central Mg_2_Cl_2_ plane.

Structure	Mg⋯μ_3_-Cl	Mg⋯μ_2_-Cl	Mg⋯μ_2_-Cl	Σθ_eq_
Title compound	2.4687 (7)	2.3825 (7)	2.3905 (8)	–
Title compound	2.4689 (7)	2.3785 (7)	2.3796 (7)	–
MGCLTF	2.789	2.398	2.405	359.6
QUJSUJ	2.888	2.405	2.406	358.0
QUJTAG	2.819	2.415	2.429	358.4
QUJTEU	2.834	2.389	2.393	356.6
SEJZUE	2.727	2.404	2.431	359.2
WILMIN	2.779	2.397	2.402	359.5

**Table 3:** Experimental details

Crystal data	
Chemical formula	[Mg_4_(C_4_H_9_)_2_Cl_6_(C_4_H_10_O)_4_]
*M*_r_	720.64
Crystal system, space group	Monoclinic, *P*2_1_/*c*
Temperature (K)	173
*a*, *b*, *c* (Å)	11.5663 (5), 15.4045 (8), 22.8256 (11)
β (°)	99.209 (4)
*V* (Å^3^)	4014.5 (3)
*Z*	4
Radiation type	Mo *K*α
μ (mm^−1^)	0.52
Crystal size (mm)	0.26 × 0.24 × 0.19

Data collection	
Diffractometer	STOE *IPDS* II two-circle-diffractometer
Absorption correction	Multi-scan (*X-AREA*; Stoe & Cie, 2001 ▸)
*T*_min_, *T*_max_	0.762, 1.000
No. of measured, independent and observed [*I* > 2σ(*I*)] reflections	20835, 7482, 5674
*R*_int_	0.033
(sin θ/λ)_max_ (Å^−1^)	0.608

Refinement	
*R*[*F* ^2^ > 2σ(*F* ^2^)], *wR*(*F* ^2^), *S*	0.030, 0.066, 0.93
No. of reflections	7482
No. of parameters	343
H-atom treatment	H-atom parameters constrained
Δρ_max_, Δρ_min_ (e Å^−3^)	0.23, −0.18

Computer programs: *X-AREA* (Stoe & Cie, 2001 ▸), *SHELXS* (Sheldrick, 2008 ▸), *SHELXL* (Sheldrick, 2015 ▸), *XP* (Sheldrick, 2008 ▸), *Mercury* (Macrae *et al.*, 2020 ▸), *PLATON* (Spek, 2009 ▸) and *publCIF* (Westrip, 2010 ▸).

## supplementary crystallographic information

### Crystal data

**Table:**

[Mg_4_(C_4_H_9_)_2_Cl_6_(C_4_H_10_O)_4_]	*F*(000) = 1536
*M**_r_* = 720.64	*D*_x_ = 1.192 Mg m^−^^3^
Monoclinic, *P*2_1_/*c*	Mo *K*α radiation, λ = 0.71073 Å
*a* = 11.5663 (5) Å	Cell parameters from 19794 reflections
*b* = 15.4045 (8) Å	θ = 3.2–25.9°
*c* = 22.8256 (11) Å	µ = 0.52 mm^−^^1^
β = 99.209 (4)°	*T* = 173 K
*V* = 4014.5 (3) Å^3^	Block, colourless
*Z* = 4	0.26 × 0.24 × 0.19 mm

### Data collection

**Table:**

STOE IPDS II two-circle-diffractometer	5674 reflections with *I* > 2σ(*I*)
Radiation source: Genix 3D IµS microfocus X-ray source	*R*_int_ = 0.033
ω scans	θ_max_ = 25.6°, θ_min_ = 3.2°
Absorption correction: multi-scan (X-Area; Stoe & Cie, 2001)	*h* = −13→14
*T*_min_ = 0.762, *T*_max_ = 1.000	*k* = −18→18
20835 measured reflections	*l* = −27→27
7482 independent reflections	

### Refinement

**Table:**

Refinement on *F*^2^	Primary atom site location: structure-invariant direct methods
Least-squares matrix: full	Hydrogen site location: inferred from neighbouring sites
*R*[*F*^2^ > 2σ(*F*^2^)] = 0.030	H-atom parameters constrained
*wR*(*F*^2^) = 0.066	*w* = 1/[σ^2^(*F*_o_^2^) + (0.0337*P*)^2^] where *P* = (*F*_o_^2^ + 2*F*_c_^2^)/3
*S* = 0.93	(Δ/σ)_max_ = 0.001
7482 reflections	Δρ_max_ = 0.23 e Å^−^^3^
343 parameters	Δρ_min_ = −0.18 e Å^−^^3^
0 restraints	

### Special details

**Table:**

Geometry. All esds (except the esd in the dihedral angle between two l.s. planes) are estimated using the full covariance matrix. The cell esds are taken into account individually in the estimation of esds in distances, angles and torsion angles; correlations between esds in cell parameters are only used when they are defined by crystal symmetry. An approximate (isotropic) treatment of cell esds is used for estimating esds involving l.s. planes.

### Fractional atomic coordinates and isotropic or equivalent isotropic displacement parameters (Å^2^)

**Table:**

	*x*	*y*	*z*	*U*_iso_*/*U*_eq_	
Mg1	0.85944 (5)	0.39400 (4)	0.67934 (3)	0.02116 (13)	
Mg2	0.55207 (5)	0.36837 (4)	0.63418 (3)	0.02481 (14)	
Mg3	0.63926 (5)	0.34063 (4)	0.79150 (3)	0.02032 (13)	
Mg4	0.94919 (5)	0.36222 (4)	0.83447 (3)	0.02314 (13)	
Cl1	0.70075 (4)	0.27496 (3)	0.69305 (2)	0.02311 (10)	
Cl2	0.69752 (4)	0.47151 (3)	0.61844 (2)	0.02928 (11)	
Cl3	1.00678 (4)	0.30899 (3)	0.74566 (2)	0.02581 (10)	
Cl4	0.79900 (4)	0.45596 (3)	0.77698 (2)	0.02215 (9)	
Cl5	0.80093 (4)	0.26496 (3)	0.85501 (2)	0.02837 (10)	
Cl6	0.48646 (4)	0.41593 (3)	0.72299 (2)	0.02612 (10)	
C1	0.42463 (17)	0.32648 (13)	0.56138 (9)	0.0304 (4)	
C2	0.3138 (2)	0.38179 (17)	0.55809 (12)	0.0535 (6)	
H2A	0.283162	0.377124	0.595581	0.080*	
H2B	0.332648	0.442554	0.551086	0.080*	
H2C	0.254675	0.361090	0.525481	0.080*	
C3	0.4687 (2)	0.33588 (19)	0.50227 (11)	0.0567 (7)	
H3A	0.491852	0.396248	0.497100	0.085*	
H3B	0.536381	0.297756	0.501815	0.085*	
H3C	0.406223	0.319693	0.469862	0.085*	
C4	0.3894 (2)	0.23210 (15)	0.56802 (12)	0.0520 (6)	
H4A	0.360749	0.224622	0.605846	0.078*	
H4B	0.327370	0.216466	0.535267	0.078*	
H4C	0.457528	0.194528	0.567220	0.078*	
C5	1.08079 (16)	0.40856 (12)	0.90376 (9)	0.0289 (4)	
C6	1.20470 (18)	0.38137 (15)	0.89651 (11)	0.0417 (5)	
H6A	1.209956	0.317874	0.896460	0.063*	
H6B	1.222861	0.404082	0.858919	0.063*	
H6C	1.260780	0.404700	0.929490	0.063*	
C7	1.0590 (2)	0.37540 (19)	0.96411 (10)	0.0529 (6)	
H7A	1.061154	0.311799	0.964420	0.079*	
H7B	1.119848	0.397862	0.995260	0.079*	
H7C	0.982052	0.395248	0.971362	0.079*	
C8	1.0774 (3)	0.50716 (15)	0.90356 (13)	0.0589 (7)	
H8A	1.091295	0.528701	0.864895	0.088*	
H8B	1.000379	0.526878	0.910870	0.088*	
H8C	1.138175	0.529492	0.934768	0.088*	
O1	0.97092 (10)	0.49894 (8)	0.67575 (6)	0.0256 (3)	
O2	0.90985 (12)	0.33409 (8)	0.60605 (6)	0.0304 (3)	
O3	0.52786 (10)	0.23817 (7)	0.80081 (6)	0.0224 (3)	
O4	0.59432 (11)	0.41160 (8)	0.86184 (6)	0.0271 (3)	
C11	1.09484 (16)	0.48893 (13)	0.67206 (10)	0.0327 (4)	
H11A	1.114637	0.526710	0.640001	0.039*	
H11B	1.109828	0.428090	0.661473	0.039*	
C12	1.1731 (2)	0.51158 (17)	0.72924 (12)	0.0499 (6)	
H12A	1.255156	0.503804	0.724439	0.075*	
H12B	1.159948	0.572157	0.739516	0.075*	
H12C	1.155139	0.473516	0.760993	0.075*	
C13	0.93368 (18)	0.58886 (12)	0.67816 (10)	0.0358 (5)	
H13A	0.991791	0.620817	0.706772	0.043*	
H13B	0.857846	0.590729	0.693006	0.043*	
C14	0.9204 (2)	0.63357 (14)	0.61938 (12)	0.0505 (6)	
H14A	0.895273	0.693650	0.623755	0.076*	
H14B	0.995619	0.633238	0.604799	0.076*	
H14C	0.861591	0.603132	0.591024	0.076*	
C21	0.94137 (19)	0.24253 (13)	0.60446 (10)	0.0379 (5)	
H21A	0.902128	0.216780	0.566729	0.045*	
H21B	0.912618	0.211830	0.637414	0.045*	
C22	1.0726 (2)	0.22900 (15)	0.60974 (12)	0.0460 (6)	
H22A	1.089483	0.166741	0.608459	0.069*	
H22B	1.101317	0.258229	0.576734	0.069*	
H22C	1.111807	0.253278	0.647419	0.069*	
C23	0.9223 (2)	0.38232 (16)	0.55274 (9)	0.0417 (5)	
H23A	1.000960	0.371057	0.542566	0.050*	
H23B	0.916738	0.445178	0.560898	0.050*	
C24	0.8313 (3)	0.3590 (2)	0.50044 (11)	0.0675 (8)	
H24A	0.843959	0.393417	0.465923	0.101*	
H24B	0.837402	0.297114	0.491483	0.101*	
H24C	0.753132	0.371277	0.509825	0.101*	
C31	0.40608 (16)	0.24950 (13)	0.80824 (10)	0.0308 (4)	
H31A	0.391010	0.311967	0.813877	0.037*	
H31B	0.392537	0.218510	0.844534	0.037*	
C32	0.3211 (2)	0.21645 (17)	0.75634 (13)	0.0540 (7)	
H32A	0.240842	0.225693	0.763725	0.081*	
H32B	0.332734	0.247793	0.720379	0.081*	
H32C	0.334263	0.154292	0.751051	0.081*	
C33	0.56613 (17)	0.14798 (11)	0.80251 (8)	0.0264 (4)	
H33A	0.641665	0.144305	0.787481	0.032*	
H33B	0.507978	0.113177	0.775802	0.032*	
C34	0.58083 (18)	0.10973 (12)	0.86411 (9)	0.0330 (4)	
H34A	0.606551	0.049237	0.862843	0.049*	
H34B	0.639597	0.143118	0.890586	0.049*	
H34C	0.505882	0.111984	0.878904	0.049*	
C41	0.57470 (18)	0.50510 (12)	0.85886 (10)	0.0318 (4)	
H41A	0.597755	0.527616	0.821778	0.038*	
H41B	0.625584	0.533061	0.892609	0.038*	
C42	0.4488 (2)	0.53006 (14)	0.86053 (11)	0.0431 (5)	
H42A	0.440950	0.593379	0.858404	0.065*	
H42B	0.397974	0.503768	0.826703	0.065*	
H42C	0.425820	0.509216	0.897577	0.065*	
C43	0.57826 (19)	0.37161 (14)	0.91760 (9)	0.0338 (4)	
H43A	0.584839	0.307827	0.913959	0.041*	
H43B	0.498345	0.384782	0.925418	0.041*	
C44	0.6661 (3)	0.40219 (19)	0.96956 (11)	0.0620 (7)	
H44A	0.651058	0.373178	1.005806	0.093*	
H44B	0.745380	0.388142	0.962586	0.093*	
H44C	0.658839	0.465138	0.974051	0.093*	

### Atomic displacement parameters (Å^2^)

**Table:**

	*U*^11^	*U*^22^	*U*^33^	*U*^12^	*U*^13^	*U*^23^
Mg1	0.0192 (3)	0.0236 (3)	0.0206 (3)	0.0005 (2)	0.0030 (2)	0.0011 (2)
Mg2	0.0200 (3)	0.0326 (3)	0.0209 (3)	−0.0027 (2)	0.0005 (2)	0.0008 (3)
Mg3	0.0190 (3)	0.0215 (3)	0.0204 (3)	0.0005 (2)	0.0032 (2)	0.0020 (2)
Mg4	0.0193 (3)	0.0290 (3)	0.0206 (3)	−0.0014 (2)	0.0016 (2)	0.0004 (2)
Cl1	0.0219 (2)	0.02278 (19)	0.0247 (2)	−0.00015 (16)	0.00402 (16)	0.00018 (17)
Cl2	0.0214 (2)	0.0340 (2)	0.0312 (3)	−0.00148 (17)	0.00056 (18)	0.01139 (19)
Cl3	0.0240 (2)	0.0295 (2)	0.0241 (2)	0.00730 (17)	0.00460 (17)	0.00182 (18)
Cl4	0.0214 (2)	0.02222 (19)	0.0231 (2)	0.00049 (15)	0.00457 (16)	0.00038 (17)
Cl5	0.0212 (2)	0.0320 (2)	0.0306 (3)	−0.00177 (17)	0.00012 (18)	0.01217 (19)
Cl6	0.0246 (2)	0.0295 (2)	0.0242 (2)	0.00760 (17)	0.00389 (17)	0.00337 (18)
C1	0.0279 (10)	0.0358 (10)	0.0259 (10)	−0.0027 (8)	−0.0004 (8)	−0.0018 (8)
C2	0.0400 (13)	0.0587 (15)	0.0559 (16)	0.0074 (11)	−0.0106 (11)	−0.0109 (13)
C3	0.0619 (17)	0.0775 (18)	0.0307 (13)	−0.0147 (14)	0.0071 (12)	−0.0069 (12)
C4	0.0538 (15)	0.0444 (13)	0.0549 (16)	−0.0087 (11)	0.0003 (12)	0.0011 (12)
C5	0.0244 (10)	0.0361 (10)	0.0247 (10)	−0.0025 (8)	−0.0008 (8)	−0.0037 (8)
C6	0.0265 (11)	0.0512 (13)	0.0453 (14)	−0.0048 (9)	−0.0005 (9)	−0.0040 (11)
C7	0.0470 (14)	0.0851 (18)	0.0252 (12)	−0.0094 (13)	0.0014 (10)	−0.0011 (12)
C8	0.0718 (18)	0.0395 (13)	0.0577 (17)	0.0032 (12)	−0.0127 (14)	−0.0152 (12)
O1	0.0195 (6)	0.0238 (6)	0.0339 (8)	−0.0012 (5)	0.0058 (5)	0.0012 (5)
O2	0.0332 (7)	0.0340 (7)	0.0252 (7)	−0.0026 (6)	0.0083 (6)	−0.0053 (6)
O3	0.0175 (6)	0.0210 (6)	0.0294 (7)	0.0000 (5)	0.0058 (5)	0.0000 (5)
O4	0.0331 (7)	0.0260 (6)	0.0235 (7)	−0.0016 (5)	0.0085 (5)	−0.0020 (5)
C11	0.0211 (10)	0.0391 (11)	0.0395 (12)	−0.0020 (8)	0.0095 (8)	0.0051 (9)
C12	0.0276 (12)	0.0617 (15)	0.0572 (16)	−0.0121 (11)	−0.0033 (10)	0.0053 (12)
C13	0.0338 (11)	0.0224 (9)	0.0506 (14)	0.0012 (8)	0.0046 (10)	−0.0013 (9)
C14	0.0425 (13)	0.0361 (12)	0.0695 (18)	−0.0088 (10)	−0.0009 (12)	0.0204 (12)
C21	0.0367 (12)	0.0360 (11)	0.0426 (13)	−0.0063 (9)	0.0109 (9)	−0.0163 (9)
C22	0.0395 (13)	0.0475 (13)	0.0536 (15)	0.0014 (10)	0.0148 (11)	−0.0190 (11)
C23	0.0416 (13)	0.0609 (14)	0.0247 (11)	−0.0049 (10)	0.0119 (9)	0.0027 (10)
C24	0.0683 (19)	0.104 (2)	0.0274 (13)	−0.0037 (16)	0.0001 (12)	−0.0077 (14)
C31	0.0188 (9)	0.0338 (10)	0.0422 (12)	0.0014 (7)	0.0125 (8)	0.0012 (9)
C32	0.0231 (11)	0.0653 (16)	0.0713 (19)	−0.0060 (10)	0.0009 (11)	−0.0117 (13)
C33	0.0317 (10)	0.0200 (8)	0.0273 (10)	0.0011 (7)	0.0045 (8)	−0.0035 (7)
C34	0.0384 (11)	0.0270 (10)	0.0327 (11)	−0.0036 (8)	0.0033 (9)	0.0045 (8)
C41	0.0363 (11)	0.0225 (9)	0.0377 (12)	−0.0029 (8)	0.0096 (9)	−0.0068 (8)
C42	0.0415 (13)	0.0384 (12)	0.0521 (15)	0.0049 (9)	0.0152 (11)	−0.0074 (10)
C43	0.0396 (12)	0.0409 (11)	0.0222 (10)	−0.0051 (9)	0.0092 (9)	−0.0017 (9)
C44	0.0760 (19)	0.0776 (19)	0.0286 (13)	−0.0134 (15)	−0.0031 (12)	−0.0065 (13)

### Geometric parameters (Å, º)

**Table:**

Mg1—O2	2.0742 (14)	O3—C31	1.456 (2)
Mg1—O1	2.0776 (13)	O3—C33	1.457 (2)
Mg1—Cl2	2.4560 (7)	O4—C43	1.453 (2)
Mg1—Cl3	2.4668 (7)	O4—C41	1.458 (2)
Mg1—Cl4	2.6204 (7)	C11—C12	1.506 (3)
Mg1—Cl1	2.6483 (7)	C11—H11A	0.9900
Mg1—Mg4	3.5608 (9)	C11—H11B	0.9900
Mg1—Mg2	3.5615 (8)	C12—H12A	0.9800
Mg2—C1	2.137 (2)	C12—H12B	0.9800
Mg2—Cl2	2.3825 (7)	C12—H12C	0.9800
Mg2—Cl6	2.3905 (8)	C13—C14	1.494 (3)
Mg2—Cl1	2.4687 (7)	C13—H13A	0.9900
Mg2—Mg3	3.5967 (9)	C13—H13B	0.9900
Mg3—O3	2.0698 (13)	C14—H14A	0.9800
Mg3—O4	2.0761 (14)	C14—H14B	0.9800
Mg3—Cl6	2.4555 (7)	C14—H14C	0.9800
Mg3—Cl5	2.4676 (7)	C21—C22	1.518 (3)
Mg3—Cl4	2.6222 (7)	C21—H21A	0.9900
Mg3—Cl1	2.6629 (7)	C21—H21B	0.9900
Mg3—Mg4	3.5774 (8)	C22—H22A	0.9800
Mg4—C5	2.135 (2)	C22—H22B	0.9800
Mg4—Cl3	2.3785 (7)	C22—H22C	0.9800
Mg4—Cl5	2.3796 (7)	C23—C24	1.503 (3)
Mg4—Cl4	2.4689 (7)	C23—H23A	0.9900
C1—C3	1.524 (3)	C23—H23B	0.9900
C1—C4	1.524 (3)	C24—H24A	0.9800
C1—C2	1.531 (3)	C24—H24B	0.9800
C2—H2A	0.9800	C24—H24C	0.9800
C2—H2B	0.9800	C31—C32	1.502 (3)
C2—H2C	0.9800	C31—H31A	0.9900
C3—H3A	0.9800	C31—H31B	0.9900
C3—H3B	0.9800	C32—H32A	0.9800
C3—H3C	0.9800	C32—H32B	0.9800
C4—H4A	0.9800	C32—H32C	0.9800
C4—H4B	0.9800	C33—C34	1.509 (3)
C4—H4C	0.9800	C33—H33A	0.9900
C5—C8	1.519 (3)	C33—H33B	0.9900
C5—C7	1.527 (3)	C34—H34A	0.9800
C5—C6	1.527 (3)	C34—H34B	0.9800
C6—H6A	0.9800	C34—H34C	0.9800
C6—H6B	0.9800	C41—C42	1.513 (3)
C6—H6C	0.9800	C41—H41A	0.9900
C7—H7A	0.9800	C41—H41B	0.9900
C7—H7B	0.9800	C42—H42A	0.9800
C7—H7C	0.9800	C42—H42B	0.9800
C8—H8A	0.9800	C42—H42C	0.9800
C8—H8B	0.9800	C43—C44	1.508 (3)
C8—H8C	0.9800	C43—H43A	0.9900
O1—C13	1.454 (2)	C43—H43B	0.9900
O1—C11	1.457 (2)	C44—H44A	0.9800
O2—C23	1.452 (2)	C44—H44B	0.9800
O2—C21	1.459 (2)	C44—H44C	0.9800
			
O2—Mg1—O1	93.33 (5)	C5—C6—H6A	109.5
O2—Mg1—Cl2	92.70 (5)	C5—C6—H6B	109.5
O1—Mg1—Cl2	91.22 (4)	H6A—C6—H6B	109.5
O2—Mg1—Cl3	90.17 (4)	C5—C6—H6C	109.5
O1—Mg1—Cl3	93.73 (4)	H6A—C6—H6C	109.5
Cl2—Mg1—Cl3	174.12 (3)	H6B—C6—H6C	109.5
O2—Mg1—Cl4	174.73 (5)	C5—C7—H7A	109.5
O1—Mg1—Cl4	90.10 (4)	C5—C7—H7B	109.5
Cl2—Mg1—Cl4	91.21 (2)	H7A—C7—H7B	109.5
Cl3—Mg1—Cl4	85.61 (2)	C5—C7—H7C	109.5
O2—Mg1—Cl1	94.41 (4)	H7A—C7—H7C	109.5
O1—Mg1—Cl1	171.76 (4)	H7B—C7—H7C	109.5
Cl2—Mg1—Cl1	85.61 (2)	C5—C8—H8A	109.5
Cl3—Mg1—Cl1	89.07 (2)	C5—C8—H8B	109.5
Cl4—Mg1—Cl1	82.38 (2)	H8A—C8—H8B	109.5
O2—Mg1—Mg4	131.76 (5)	C5—C8—H8C	109.5
O1—Mg1—Mg4	93.56 (4)	H8A—C8—H8C	109.5
Cl2—Mg1—Mg4	134.76 (3)	H8B—C8—H8C	109.5
Cl3—Mg1—Mg4	41.745 (17)	C13—O1—C11	113.76 (14)
Cl4—Mg1—Mg4	43.883 (16)	C13—O1—Mg1	123.38 (11)
Cl1—Mg1—Mg4	83.37 (2)	C11—O1—Mg1	122.84 (11)
O2—Mg1—Mg2	96.53 (4)	C23—O2—C21	114.54 (16)
O1—Mg1—Mg2	132.21 (4)	C23—O2—Mg1	121.76 (13)
Cl2—Mg1—Mg2	41.808 (17)	C21—O2—Mg1	123.63 (12)
Cl3—Mg1—Mg2	132.72 (2)	C31—O3—C33	113.99 (13)
Cl4—Mg1—Mg2	84.07 (2)	C31—O3—Mg3	123.39 (10)
Cl1—Mg1—Mg2	43.851 (16)	C33—O3—Mg3	122.58 (10)
Mg4—Mg1—Mg2	113.03 (2)	C43—O4—C41	114.78 (15)
C1—Mg2—Cl2	120.00 (6)	C43—O4—Mg3	122.50 (11)
C1—Mg2—Cl6	118.61 (6)	C41—O4—Mg3	122.72 (11)
Cl2—Mg2—Cl6	104.54 (3)	O1—C11—C12	112.69 (18)
C1—Mg2—Cl1	125.52 (6)	O1—C11—H11A	109.1
Cl2—Mg2—Cl1	91.35 (2)	C12—C11—H11A	109.1
Cl6—Mg2—Cl1	90.28 (3)	O1—C11—H11B	109.1
C1—Mg2—Mg1	142.27 (6)	C12—C11—H11B	109.1
Cl2—Mg2—Mg1	43.408 (17)	H11A—C11—H11B	107.8
Cl6—Mg2—Mg1	99.10 (2)	C11—C12—H12A	109.5
Cl1—Mg2—Mg1	48.005 (17)	C11—C12—H12B	109.5
C1—Mg2—Mg3	142.07 (6)	H12A—C12—H12B	109.5
Cl2—Mg2—Mg3	97.92 (2)	C11—C12—H12C	109.5
Cl6—Mg2—Mg3	42.779 (17)	H12A—C12—H12C	109.5
Cl1—Mg2—Mg3	47.751 (17)	H12B—C12—H12C	109.5
Mg1—Mg2—Mg3	67.468 (17)	O1—C13—C14	113.18 (19)
O3—Mg3—O4	94.89 (5)	O1—C13—H13A	108.9
O3—Mg3—Cl6	91.67 (4)	C14—C13—H13A	108.9
O4—Mg3—Cl6	90.00 (4)	O1—C13—H13B	108.9
O3—Mg3—Cl5	90.29 (4)	C14—C13—H13B	108.9
O4—Mg3—Cl5	93.12 (4)	H13A—C13—H13B	107.8
Cl6—Mg3—Cl5	176.16 (3)	C13—C14—H14A	109.5
O3—Mg3—Cl4	172.94 (4)	C13—C14—H14B	109.5
O4—Mg3—Cl4	90.55 (4)	H14A—C14—H14B	109.5
Cl6—Mg3—Cl4	92.83 (2)	C13—C14—H14C	109.5
Cl5—Mg3—Cl4	84.91 (2)	H14A—C14—H14C	109.5
O3—Mg3—Cl1	92.96 (4)	H14B—C14—H14C	109.5
O4—Mg3—Cl1	170.55 (4)	O2—C21—C22	112.44 (16)
Cl6—Mg3—Cl1	84.50 (2)	O2—C21—H21A	109.1
Cl5—Mg3—Cl1	92.10 (2)	C22—C21—H21A	109.1
Cl4—Mg3—Cl1	82.07 (2)	O2—C21—H21B	109.1
O3—Mg3—Mg4	130.97 (4)	C22—C21—H21B	109.1
O4—Mg3—Mg4	95.91 (4)	H21A—C21—H21B	107.8
Cl6—Mg3—Mg4	135.91 (3)	C21—C22—H22A	109.5
Cl5—Mg3—Mg4	41.493 (17)	C21—C22—H22B	109.5
Cl4—Mg3—Mg4	43.636 (16)	H22A—C22—H22B	109.5
Cl1—Mg3—Mg4	82.84 (2)	C21—C22—H22C	109.5
O3—Mg3—Mg2	96.51 (4)	H22A—C22—H22C	109.5
O4—Mg3—Mg2	130.14 (4)	H22B—C22—H22C	109.5
Cl6—Mg3—Mg2	41.392 (17)	O2—C23—C24	113.1 (2)
Cl5—Mg3—Mg2	135.04 (3)	O2—C23—H23A	109.0
Cl4—Mg3—Mg2	83.34 (2)	C24—C23—H23A	109.0
Cl1—Mg3—Mg2	43.334 (16)	O2—C23—H23B	109.0
Mg4—Mg3—Mg2	111.79 (2)	C24—C23—H23B	109.0
C5—Mg4—Cl3	118.88 (6)	H23A—C23—H23B	107.8
C5—Mg4—Cl5	121.03 (6)	C23—C24—H24A	109.5
Cl3—Mg4—Cl5	105.06 (3)	C23—C24—H24B	109.5
C5—Mg4—Cl4	123.76 (6)	H24A—C24—H24B	109.5
Cl3—Mg4—Cl4	91.02 (2)	C23—C24—H24C	109.5
Cl5—Mg4—Cl4	90.29 (2)	H24A—C24—H24C	109.5
C5—Mg4—Mg1	138.64 (6)	H24B—C24—H24C	109.5
Cl3—Mg4—Mg1	43.673 (17)	O3—C31—C32	112.99 (17)
Cl5—Mg4—Mg1	100.25 (2)	O3—C31—H31A	109.0
Cl4—Mg4—Mg1	47.368 (17)	C32—C31—H31A	109.0
C5—Mg4—Mg3	142.99 (6)	O3—C31—H31B	109.0
Cl3—Mg4—Mg3	98.01 (2)	C32—C31—H31B	109.0
Cl5—Mg4—Mg3	43.395 (17)	H31A—C31—H31B	107.8
Cl4—Mg4—Mg3	47.132 (17)	C31—C32—H32A	109.5
Mg1—Mg4—Mg3	67.685 (17)	C31—C32—H32B	109.5
Mg2—Cl1—Mg1	88.14 (2)	H32A—C32—H32B	109.5
Mg2—Cl1—Mg3	88.92 (2)	C31—C32—H32C	109.5
Mg1—Cl1—Mg3	96.92 (2)	H32A—C32—H32C	109.5
Mg2—Cl2—Mg1	94.78 (3)	H32B—C32—H32C	109.5
Mg4—Cl3—Mg1	94.58 (2)	O3—C33—C34	112.69 (15)
Mg4—Cl4—Mg1	88.75 (2)	O3—C33—H33A	109.1
Mg4—Cl4—Mg3	89.23 (2)	C34—C33—H33A	109.1
Mg1—Cl4—Mg3	98.63 (2)	O3—C33—H33B	109.1
Mg4—Cl5—Mg3	95.11 (2)	C34—C33—H33B	109.1
Mg2—Cl6—Mg3	95.83 (2)	H33A—C33—H33B	107.8
C3—C1—C4	108.14 (19)	C33—C34—H34A	109.5
C3—C1—C2	107.4 (2)	C33—C34—H34B	109.5
C4—C1—C2	107.51 (19)	H34A—C34—H34B	109.5
C3—C1—Mg2	111.90 (15)	C33—C34—H34C	109.5
C4—C1—Mg2	111.89 (15)	H34A—C34—H34C	109.5
C2—C1—Mg2	109.76 (14)	H34B—C34—H34C	109.5
C1—C2—H2A	109.5	O4—C41—C42	113.16 (16)
C1—C2—H2B	109.5	O4—C41—H41A	108.9
H2A—C2—H2B	109.5	C42—C41—H41A	108.9
C1—C2—H2C	109.5	O4—C41—H41B	108.9
H2A—C2—H2C	109.5	C42—C41—H41B	108.9
H2B—C2—H2C	109.5	H41A—C41—H41B	107.8
C1—C3—H3A	109.5	C41—C42—H42A	109.5
C1—C3—H3B	109.5	C41—C42—H42B	109.5
H3A—C3—H3B	109.5	H42A—C42—H42B	109.5
C1—C3—H3C	109.5	C41—C42—H42C	109.5
H3A—C3—H3C	109.5	H42A—C42—H42C	109.5
H3B—C3—H3C	109.5	H42B—C42—H42C	109.5
C1—C4—H4A	109.5	O4—C43—C44	113.08 (18)
C1—C4—H4B	109.5	O4—C43—H43A	109.0
H4A—C4—H4B	109.5	C44—C43—H43A	109.0
C1—C4—H4C	109.5	O4—C43—H43B	109.0
H4A—C4—H4C	109.5	C44—C43—H43B	109.0
H4B—C4—H4C	109.5	H43A—C43—H43B	107.8
C8—C5—C7	109.2 (2)	C43—C44—H44A	109.5
C8—C5—C6	107.36 (19)	C43—C44—H44B	109.5
C7—C5—C6	107.40 (18)	H44A—C44—H44B	109.5
C8—C5—Mg4	108.47 (15)	C43—C44—H44C	109.5
C7—C5—Mg4	110.82 (14)	H44A—C44—H44C	109.5
C6—C5—Mg4	113.46 (14)	H44B—C44—H44C	109.5
			
C13—O1—C11—C12	71.5 (2)	C33—O3—C31—C32	−68.9 (2)
Mg1—O1—C11—C12	−107.09 (18)	Mg3—O3—C31—C32	113.14 (17)
C11—O1—C13—C14	76.2 (2)	C31—O3—C33—C34	−72.6 (2)
Mg1—O1—C13—C14	−105.27 (18)	Mg3—O3—C33—C34	105.41 (16)
C23—O2—C21—C22	72.4 (2)	C43—O4—C41—C42	−66.1 (2)
Mg1—O2—C21—C22	−104.48 (19)	Mg3—O4—C41—C42	112.98 (17)
C21—O2—C23—C24	71.3 (3)	C41—O4—C43—C44	−64.8 (2)
Mg1—O2—C23—C24	−111.7 (2)	Mg3—O4—C43—C44	116.12 (19)

### Hydrogen-bond geometry (Å, º)

**Table:**

*D*—H···*A*	*D*—H	H···*A*	*D*···*A*	*D*—H···*A*
C32—H32*A*···Cl3^i^	0.98	2.96	3.876 (2)	155
C34—H34*A*···Cl6^ii^	0.98	2.92	3.602 (2)	127

Symmetry codes: (i) *x*−1, *y*, *z*; (ii) −*x*+1, *y*−1/2, −*z*+3/2.
